# Investigation of Tactile Perception Evoked by Ridged Texture Using ERP and Non-linear Methods

**DOI:** 10.3389/fnins.2021.676837

**Published:** 2021-06-24

**Authors:** Wei Tang, Meimei Zhang, Guofang Chen, Rui Liu, Yuxing Peng, Si Chen, Yibing Shi, Chunai Hu, Shengjie Bai

**Affiliations:** ^1^School of Mechatronic Engineering, China University of Mining and Technology, Xuzhou, China; ^2^Xuzhou Central Hospital, Xuzhou, China; ^3^Fluid Machinery Center, Jiangsu University, Zhenjiang, China

**Keywords:** tactile perception, ERP components, recurrence plots, recurrence quantification analysis, ridged texture

## Abstract

The triangular ridged surface can improve the grip reliability of products, but the sharp edge of triangular ridge induces sharp and uncomfortable feeling. To study the effect of edge shape (sharp, round, and flat shape) of triangular ridges on brain activity during touching, electroencephalograph (EEG) signals during tactile perception were evaluated using event-related potentials (ERP) and non-linear analysis methods. The results showed that the early component of P100 and P200, and the late component of P300 were successfully induced during perceiving the ridged texture. The edge shape features affect the electrical activity of brain during the tactile perceptions. The sharp shape feature evoked fast P100 latency and high P100 amplitude. The flat texture with complex (sharp and flat) shape feature evoked fast P200 latency, high P200 amplitude and RQA parameters. Both of the sharp shape and complex shape feature tended to evoke high peak amplitude of P300. The large-scale structures of recurrence plots (RPs) and recurrence quantification analysis (RQA) parameters can visually and quantitatively characterize the evolution regulation of the dynamic behavior of EEG system along with the tactile process. This study proved that RPs and RQA were protential methods for the feature extraction and state recognition of EEG during tactile perception of textured surface. This research contributes to optimize surface tactile characteristics on products, especially effective surface textures design for good grip.

## Introduction

Tactile perception of products is of great interest to engineers and product designers since suitable surface characteristics can improve their function or to please the user. Studies showed that texture designs, either triangular or rectangular ridge, are effective methods to increase the friction and therefore improve the grip of user, such as on sports equipment, tool handles, phone cases, and power tools (Tomlinson et al., 2011, 2013; Darden and Schwartz, 2013; Wilde and Schwartz, 2016). The parallel ridge textures influenced the frictional characteristics of the surface based on the geometry parameters of the ridges. The influence of simple textures on skin friction and the frictional mechanism are well investigated (Zhang et al., 2015; Ben Messaoud et al., 2016; Tang et al., 2020a). However, in addition of friction, the study of haptics should also focus on understanding the neurophysiology interactions that take place during touching (Fagiani and Barbieri, 2016). It is difficult to evaluate and to predict tactile perception according to just the friction behavior of skin.

Since tactile perception is processed in the cerebral cortex of the brain, the cortical activity related to tactile perception is technically challenging. An increasing number of functional magnetic resonance imaging (fMRI) studies showed the neural correlates for tactile perception of macro geometric properties, material properties, and object recognition itself (Kitada, 2016; Sathian, 2016), for example the thermal perception (Craig et al., 2000), softness perception (Servos et al., 2001; Kitada et al., 2019), and stickiness perception (Yeon et al., 2017). EEG with high temporal resolution and low cost of use is one of the most-used technologies for the examination of these cortical activities. EEG and ERP have been widely used for assessing cognitive functions and brain ability. Different natural materials, including fabrics and paper, were commonly chosen to investigate the physiological responses of the brain in the process of tactile perception by EEG and ERP (Moungou et al., 2016; Camillieri et al., 2018). Some studies showed that the vibration and friction during tactile perception can elicit a neuronal entrainment at the cortical level, appearing as peaks of the EEG frequency spectrum (Camillieri et al., 2018), amplitude, and peak latency of P300 component (Chen and Ge, 2017; Tang et al., 2020b). Previous study about perceptual processing also reported the earlier somatosensory evoked potential (SEP) components within or around 100 ms (Inui et al., 2004; Adhikari et al., 2014). These components extracted from the EEG and ERP proved that they can be used to isolate and to explore the brain activity related to the tactile exploration of natural textures (Moungou et al., 2016). However, due to the wide range and diversity of factors that have been found to affect these components, the theory of the underlying neural processes is difficult to know.

Traditional EEG analysis methods mainly focus on the linear time-domain and frequency-domain features. However, the electrical activity of the brain measured by EEG is a comprehensive potential of a large number of neurons in the cortex. Neurons are known to be non-linear devices since a certain threshold has to be crossed for the neuron to fire (Kandel et al., 1996). EEG signals exhibit strong non-linear and dynamic nature (Natrajana et al., 2004). In this sense, the non-linear method is a better method than traditional linear methods in characterizing the intrinsic nature of EEG, understanding the underlying brain processes during tactile perception, and searching for their physiological significance. Some non-linear dynamical parameters, such as correlation dimension, Lyapunov index, wavelet entropy, and recursive analysis, have been used in the study of EEG signals (Rombouts et al., 1995; Lamberts et al., 2000; Natrajana et al., 2004). RPs and the RQA method, which are based on the relative distance between phase points, are widely used in the analysis of non-linear systems. RPs can be used to observe the non-linear time series internal dynamic mechanism of recursive phenomenon, and RQA can quantify the recurrence feature of RPs. Meanwhile, compared with other methods, RPs are more suitable for the non-linear analysis of EEG data since they require less steady-state performance of the signal and are not easily affected by signal noise (Marwan and Meinke, 2004; Schinkel et al., 2007; Acharya et al., 2011).

According previous tactile friction studies, though the triangular ridged surface can improve the grip reliability of products (Tomlinson et al., 2011, 2013), but the sharp edge of triangular ridge induces sharp and uncomfortable feeling. In order to compare the effects of edge shape of triangular ridge on brain activity during touching, except the sharp edge of triangular ridge, the sharp edge was also fileted and chamfered to obtain different edge shape. EEG signals of subjects when they performed a tactile task with ridged texture were discussed using ERP and non-linear methods. The evolution of the non-linear dynamic behavior of EEG systems related with tactile perception was characterized quantitatively. This study can be coupled with comfort and tactile friction studies to optimize surface tactile characteristics on products, especially effective surface textures design for good grip. It also provides quantitative evaluation methods for skin-touched products and surfaces, such as skin cream and fabrics since the commercial competitiveness of these products is strongly related to their tactile feeling and to their acceptance by the hand (Fagiani and Barbieri, 2016).

## Experimental Methods

### Test Bed

**FIGURE 1:**
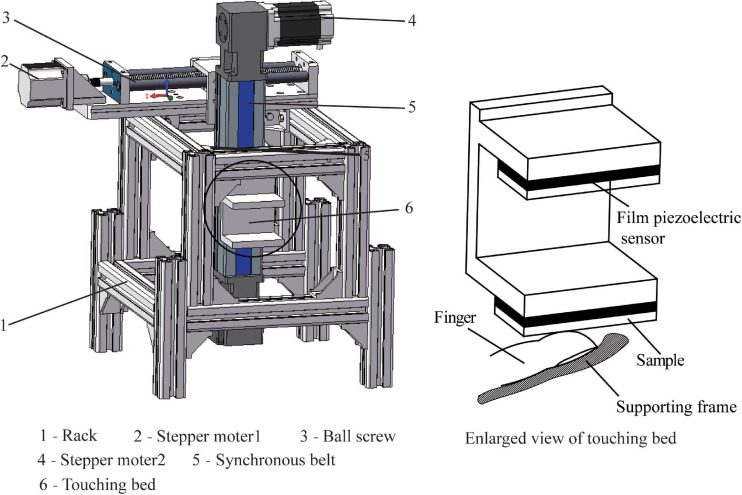
Structure schematic diagram of test bed.

A test bed was designed for the EEG/ERP (event-related potential) experiment. As shown in Figure 1, the test bed is mainly composed of the rack, motion controller component, sample holder, and a synchronous trigger system. The motion controller component included a single-chip microcomputer, a driver, a keypad, a stepping motor, a synchronous wheel, a synchronous belt, a ball screw, and coupling, and it was used to control the “up-and-down” and “reciprocating” motion of the sample holder. Two thin film piezoelectric sensors were fixed on the two stages of the sample holder. The samples were attached on the thin film piezoelectric sensor by double-coating tape. Once the finger touched the sample surface, the touching normal load was changed into the electric signal by the film sensor that triggered the EEG system amplifier and marked the start of the touch. In this way, the synchronization of the touching action and EEG data collection could be ensured. In addition, the piezoelectric film sensor can monitor the touching load and ensure it remains approximately consistent for each touch. A supporting frame for finger was designed to increase the comfortability and positional stability of the finger.

### Samples

**FIGURE 2:**
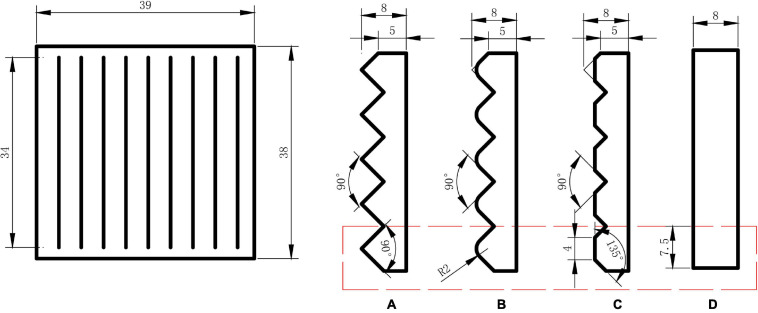
Samples with ridged textures of **(A)** sharp shape, **(B)** round shape, **(C)** flat shape, and **(D)** smooth sample.

In the ERP test, a sample without any texture was selected as the non-target sample. Samples with different ridged texture (sharp shape, round shape, and flat shape) were chosen as the target sample of the tactile stimulus, which are shown in Figure 2. The ridged texture was designed as following method: the sharp edge of sharp sample was fileted to obtain the round shape sample; the sharp edge of sharp sample was chamfered to obtain the flat shape sample. The sample materials were acrylic plates with Young’s modulus of 3000 MPa and Poisson coefficient of 0.39. The ridged textures of the samples were made by laser processing.

### Stimuli

An Oddball classic paradigm was used to evoke the ERP components. Oddball usually requires two of the same type of stimuli simultaneously, namely, the target and non-target stimuli (Teodorescu and Rahnejat, 2008). In this study, the non-target sample occurred 75% of the time, and target samples occurred 25% of the time. The sample test order used a pseudorandom sequence that was not revealed to the subjects, which included a total of 120 stimulations (30 times for the target stimulation and 90 times for the non-target stimulation). Only when the subject actively participated in the task, could the significant ERP waveform be induced. To make the subject focus on the test, the subjects were required to click the mouse button once to feel the target sample.

The experiment consisted of the three groups listed in Table 1. The touch trial was controlled by single-chip programming. Specifically, once the sample touches the finger, it slides along the finger surface with a constant normal load approximately 0.8 N and a 10 mm sliding distance for 1.0 s and then takes 2 s to reset. There were 120 touches for one trial. Each touch trial took approximately 6 min and was repeated twice with 5 min rest. The resting time between each group test was 15 min. The total duration of the experiment was 81 min.

**TABLE 1:** Test groups.

	**Non-target sample**	**Target sample**
Group 1	Smooth	Sharp shape
Group 2	Smooth	Round shape
Group 3	Smooth	Flat shape

### Participants

Twelve, right-handed males who were aged 19 to 24 years (mean ± standard deviation = 21.6 ± 1.5 years) participated in this study. None of the subjects had histories of tactile impairments, neurological disorders, or psychological disorders. Personal information was obtained from each of the participants before the experiment. This study was conducted under the guidance of the international ethical standards and was approved by Ethics Committee of Xuzhou Centre Hospital (No. XZXY-LJ-20210513-054). All of the subjects provided informed written consent.

The volunteers washed and dried their hair and then relaxed for 20 min in an experiment room at 24–26° and 40–60% relative humidity. The right index finger was selected as the test position. To avoid the influence of body movement during touching, a passive touching method was adopted. The subjects sat in a comfortable position and placed their arms and fingers on the rack with their ears plugged. Volunteers adjusted the position of the fingers and let the normal load remain approximately 0.8 N, which was monitored by the film piezoelectric sensors. Then, the fingers were fixed by the tap.

### EEG Acquisition Equipment

Electroencephalograph data were recorded by a 32-channel EEG-System (ANT Neuro, Hengelo, Netherlands) with sampling frequency of 1000 Hz. Electrode impedance was maintained below 5 kΩ throughout the study.

**FIGURE 3:**
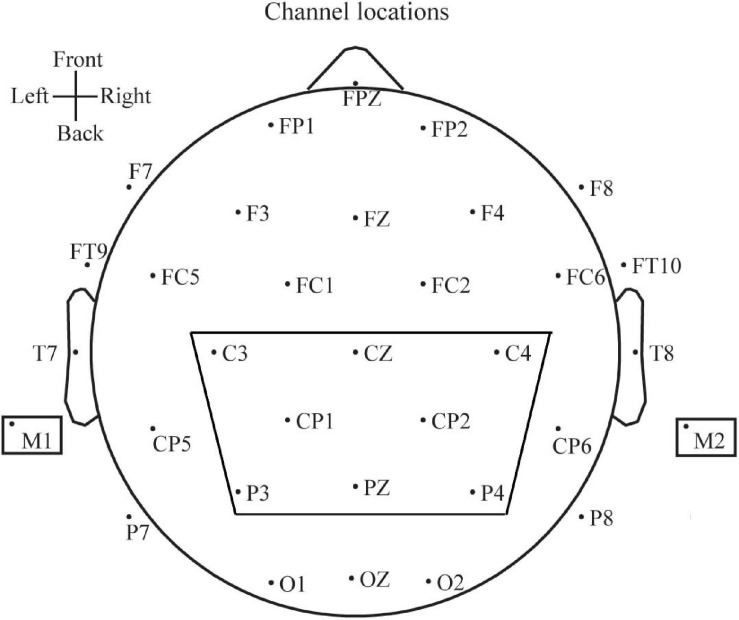
Electrode distribution pattern.

Since the parietal lobe is the part of the brain that is most strongly related to tactile perception (Gottlieb, 2007; Chen et al., 2019), we selected C3, CP1, P3, CZ, PZ, C4, CP2, and P4 electrodes for further analysis, as shown in Figure 3. The M1 and M2 earlobe electrodes were chosen as reference electrodes.

### ERP Data Analysis Methods

Electroencephalograph data were processed through the EEGLAB, which is an open source MATLAB toolbox for physiological research. The data were referenced to an averaged montage of ears, the bandpass was filtered between 0.5 Hz and 40 Hz, and the baseline correction was done. Then, the target stimulus data were sectioned and extracted for the feature extraction. The ERP waveform was obtained by superimposing and averaging the EEG data of all target stimulus in one trial. Two participants failed to induce the ERP waveform. We excluded two participants from the data analysis. As a result, data analyses were performed on 10 participants out of 12 in total.

#### ERP Analysis

The latency and amplitude of ERP wave represent the length of time spent and the amount of neural resources participating during information processing (Lin et al., 2019). An ERP component is typically labeled according to its polarity and its peak latency in the waveform. In this study, P100, P200, and P300 components of ERP waveform were analyzed.

The P100 and P200 are early ERP components, and P300 is the late ERP components. They are positive going electrical potential that peaks at about 100 ms (or within 100 ms), 200 ms (varying between about 150 and 275 ms), and 300 ms (varying between about 250 and 500 ms) after the onset of some external stimulus. These potential changes measurable from the scalp are believed to reflect the post-synaptic activity of a specific neural process. The P100 and P200 are considered to be the exogenous potential, since they are sensitive to physical features of the evoking stimuli. They reflect changes in perceptual-level processing rather than decision-level processing (Schupp et al., 2004) and was believed to signify attentional recruitment and modulates perceptual processing (Bourisly and Shuaib, 2018). The P300 component of ERP is a typical endogenous component elicited in the process of decision making that reflects the basic cognitive processes.

Thus, a program in MATLAB was designed to obtain the peak voltage of the ERP waveform in the time window 60–120, 170–200, and 260–300 ms for each electrode, and this was chosen as the P100, P200, and P300 peak amplitude. The time corresponding to the peak voltage was chosen as the corresponding latency. The average peak amplitude and latency of ERP components for all trials for each electrode were calculated.

All data were evaluated with SPSS statistics software. Shapiro–Wilk test was used for normality test. The results show all data follow normal distribution. One-way analysis of variance (ANOVA) was used to determine whether there are any statistically significant differences of the means of latency and amplitude of ERP components between three texture shape groups, then Bonferroni-adjusted significance tests was used for pairwise comparisons. Differences at *P* < 0.05 were considered to be statistically significant.

#### Non-linear Analysis

In this study, in order to characterize the evolution of the non-linear dynamic behavior of ERP evoked by tactile perception quantitatively, RPs, and RQA were explored to analyze the non-linear dynamic recurrence characteristics of tactile ERP signals induced by different texture shapes.

Since the recursive features of non-linear time series signal are mapped on the phase space, the phase space reconstruction should be carried out before the recursive analysis of the signal. According to the time delay embedding technique proposed by Takens (1981), the phase space can be reconstructed by using a time delay embedding as follows:

(1) $$\dot{\vec{x}\left(i\right)=(u\left(i\right),u\left(i+\tau \right),\cdots ,u\left(i+\tau \left(m-1\right)\right)}$$

where, $\vec{x}\,\left(i\right)$ is the phase space trajectory*u*(*i*) is time series, *m* is the embedding dimension and *τ* is the time delay. In this study, *τ* and *m* were chosen as 18 and 5 established by the Mutual Information (MI) method and False Nearest Neighbors (FNN) method, respectively.

As a non-linear analysis technique, RPs are used to visualize the recurrence behavior of the phase space trajectory$\vec{x}\,\left(i\right)$ of dynamical systems. The main step of this visualization is the calculation of a binary recurrence matrix *R* in which the matrix elements correspond to those times at which the trajectory is at the same place, i.e., the set of *(i, j)* with $\vec{x}\,\left(i\right)=\vec{x}\,\left(j\right)$. The entry *R* is expressed as follows:

(2) $$R\,\left(i,j\right)=\left\{ \begin{array}{c} 1\;i\,f\,||\vec{x}\,\left(i\right)-\vec{x}\,\left(j\right)||\leq \varepsilon \\ 0\;\text{otherwise} \end{array}\right.$$

where, *R (i, j)* is an element of the recurrence matrix; ε is the threshold distance. According to Zbilut et al. (2002), ε was selected to make the recurrence rate equal to 0.3. RPs were marked as black dots at coordinates *(i, j)* if *R (i, j)* = 1. RPs mostly contain single dots and lines (continuous dots), which are parallel to the mean diagonal (line of identity, LOI) or which are vertical/horizontal.

Recurrence quantification analysis is a non-linear data analysis method used to quantify the RPs in order to explore dynamical systems, based on the small-scale structures therein. In this study, longest vertical line *V*_max_, mean diagonal line length *L*, mean length of the vertical lines *TT*, and Shannon entropy *ENT* showed significant differences and were chosen to quantize the dynamic characteristics of EEG during tactile perception.

Longest vertical line *V*_max_ is the maximum length of the vertical lines and is given by:

(3) V=maxmax({vi;i=1⋯N}v)

The mean diagonal line length *L* is the average length of the lines parallel to LOI and quantifies the mean prediction time or the inverse of the divergence of the system (Marwan et al., 2007). It is defined as follows:

(4) $$L=\frac{\sum_{l=l_{\text{min}}}^{N}l\,P\,\left(l\right)}{\sum_{l=l_{\text{min}}}^{N}P\,\left(l\right)}$$

where, *P*(*l*) is the frequency distribution of the lengths *l* of the diagonal lines, which have at least a length of *l*_min_.

The trapping time *TT* measured the mean length of the vertical lines and is related with the laminarity time of the dynamical system. It is given by as follows:

(5) $$T\,T=\frac{\sum_{v=v_{\text{min}}}^{N}v\,P\,\left(v\right)}{\sum_{v=v_{\text{min}}}^{N}P\,\left(v\right)}$$

where, *P(υ*) is the frequency distribution of the lengths *υ* of the vertical lines, which have at least a length of *υ_min_*.

The Shannon entropy *ENT* of *P*(*l*) reflects the complexity of the recurrence structure. It is given as follows:

(6) E⁢N⁢T=-∑pl=lminN⁢(l)⁢l⁢n⁢p⁢(l)

## Results and Discussion

### Shape Features of Ridged Texture

The shape features of one ridge element (marked with red rectangle in Figure 2) were shown in Table 2. The results show that the edge angle of sharp shape texture was 90° which has sharp feature and the round shape texture has 2 mm filet which shows round feature. The flat shape texture is formed by 135° edge and 4 mm flat, which has both sharp and flat feature, namely complex shape feature. Compared with other three textures, flat shape sample has more complex geometric structure.

**TABLE 2:** Shape features of ridge element.

**Shape features**	**Target samples**			**Non-target sample**
	**Sharp shape**	**Round shape**	**Flat shape**	**Smooth surface**
Angle of edge	90°	–	135°	–
Radius of edge	–	2 mm	–	–
Flat length	–	–	4 mm	7.5 mm

### ERP Analysis

**FIGURE 4:**
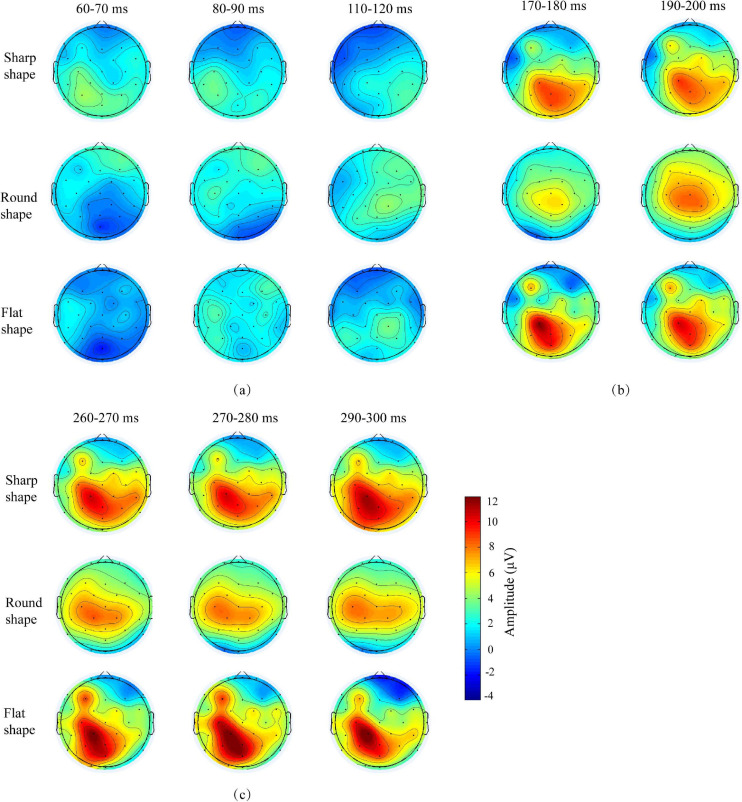
Average ERP scalp maps of three shapes of texture samples from 0 to 600 ms.

Figure 4 shows the average ERP scalp maps of 32 electrodes at the time window of 60–120, 170–200, and 260–300 ms for the three samples. The color change from red to blue corresponds to the voltage change from high to low. At 60–70 ms, the exogenous component P100 was evoked and the tactile perception of the texture was sensed by the cerebral cortex. Before 90 ms, the scalp topography was mainly located in the left hemisphere, indicating the activity of neuron was focused on the contralateral to the side of stimulation, in close agreement with previous studies that generators mainly located in contralateral primary somatosensory cortex during this time (Lucan et al., 2010). After 110 ms, the scalp topography was mainly bilateral that was consistent with intracranial evidence for bilateral contributions from secondary somatosensory areas after 100 ms (Allison et al., 1992; Frot and Mauguiere, 1999). The scalp topography also showed that the parietal lobe area was positively activated, which is responsible for recognizing the shapes and textures of objects according to Gottlieb (2007) and Chen et al. (2019). Our results are consistent with these reports.

**FIGURE 5:**
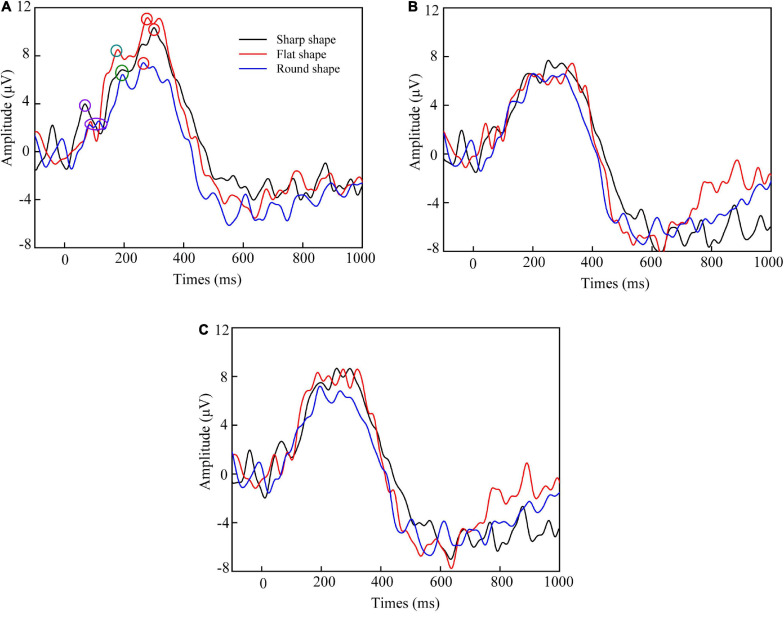
Average ERP waveform of **(A)** C3, CP1, and P3 electrodes located in the left hemisphere, **(B)** C4, CP2, and P4 located in the right hemisphere, and **(C)** CZ and PZ located in the middle hemisphere. The purple, green, and red circle corresponded to the P100, P200, and P300 peak, respectively.

Figure 5 shows the average ERP waveform of the chosen electrodes. The scalp topography and ERP waveform showed that the ERP amplitude of contralateral area was significantly higher than those in the right and middle hemisphere. This conforms to the rule that the sensation of mechanical stimulation usually occurs primarily in the contralateral brain. So the C3, CP1, and P3 electrodes located in the left hemisphere were further analyzed in the non-linear analysis.

Table 3 summarized the mean latency and amplitude of ERP components. The results show that the early component of P100 (60–120 ms) and P200 (170–200 ms), and the late component of P300 (260–300 ms) were successfully induced during perceiving the three target samples. Previous study about perceptual processing also reported earlier somatosensory evoked potential (SEP) components (within or around 100 ms) following tactile stimulation (Inui et al., 2004; Lucan et al., 2010; Adhikari et al., 2014). According to these studies, the early response originates from the primary somatosensory cortex (SI) and is highly representative of the stimulus characteristics (for example intensity and frequency).

**TABLE 3:** Summary of the latency and amplitude of ERP components.

	**Latency (ms)/amplitude (μV)**		
	**P100**	**P200**	**P300**
Sharp shape	67^b^/4.3^b^	192^a^/6.9^a^	299^b^/10.6^a^
Round shape	80^a^/2.4^a^	195^a^/6.7^a^	264^a^/7.5^b^
Flat shape	86^a^/2.8^a^	179^b^/8.5^b^	274^a^/11.2^a^
*P*	0.002/0	0.023/0	0/0

*Means values were calculated from all samples. Means in a line sharing different letter are significantly different and sharing same letter are not significantly different for each shape sample (*P* < 0.05).*

Event-related potentials early components have been well-characterized in studies that focus primarily on visual sensation. P100 is usually interpreted as a neurophysiological indicator of preferential attention to sensory inputs (Key et al., 2005). The amplitude and latency of the P100 cortical evoked potential are governed solely by properties intrinsic to the stimulus. In this study, the results showed that the sharp shape texture induced the faster P100 latency of 67 ms and larger P100 amplitude of 4.3 μV, which were significantly different with other shape textures at *P* < 0.05. It suggested that the sharp shape feature was preferentially perceived. It was explained that the sharp feature of texture increased the contact pressure and skin deformation. The cutaneous mechanoreceptors input the strong tactile stimulation to the corresponding sensing area of cerebral cortex. The sharp feature of texture promoted the neurotransmission, which in-turn enhanced the neuronal response properties and temporal processing.

The results also showed that the flat shape texture induced the faster P200 latency of 179 ms and larger P200 amplitude of 8.5 μV, which were significantly different with other shape textures at *P* < 0.05. According to Table 2, the flat shape has more complex geometric structure, so that the subject can sense more often the change of shape than others, which in-turn enhanced the neuronal response properties and temporal processing.

The P300 component of ERP is elicited in the process of decision making that reflects the basic cognitive processes. The peak amplitude of P300 showed the attention of volunteers’ judgment of the texture shape. According to Gray et al. (2004), the P300 peak amplitude is proportional to the amount of attentional resources engaged in processing a given stimulus. The results showed that the peak amplitude of P300 evoked by round shape texture was significantly lower than others (*P* < 0.05). It is because compared with smooth surface, the sharp feature of sharp shape sample and complex (sharp and flat) shape feature of flat shape sample can be sensed easily and need less attentional resources in the tactile perception judgment.

### Non-linear Analysis

**FIGURE 6:**
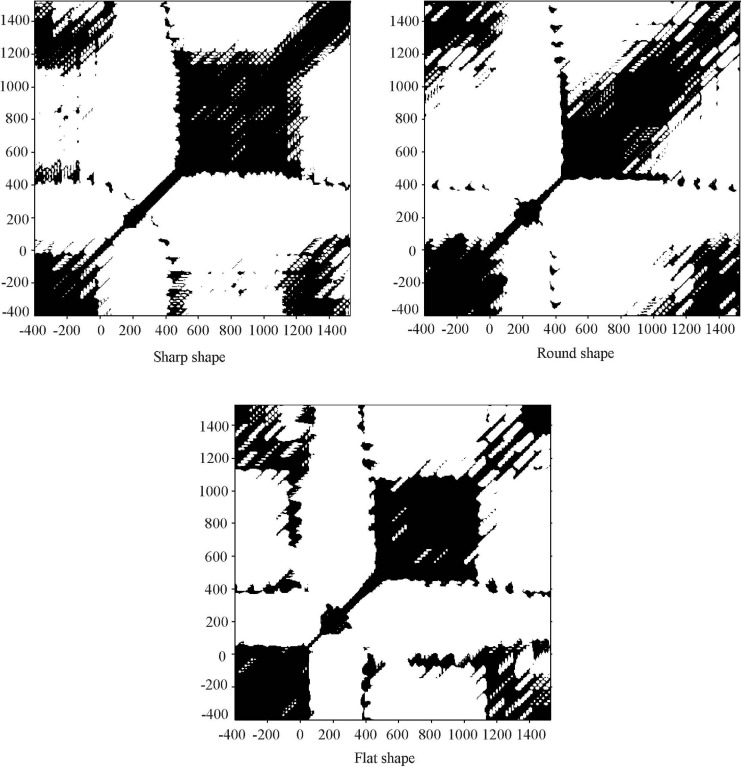
Recurrence plots of ERP signals stimulated by three samples.

**FIGURE 7:**
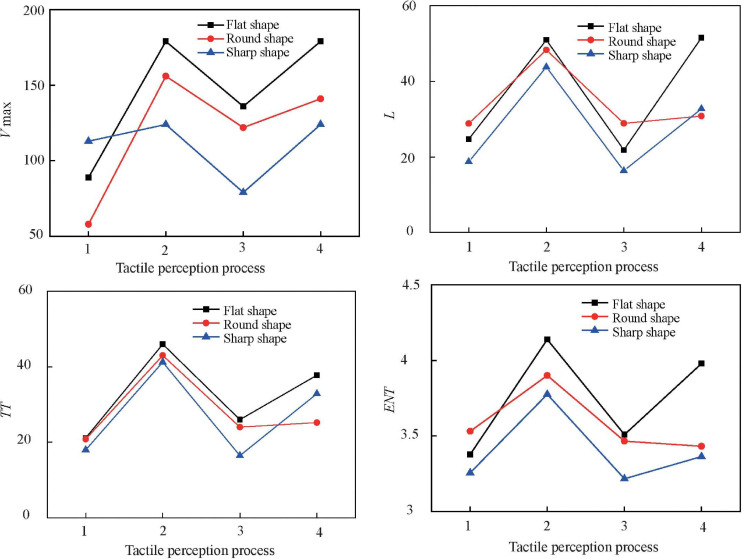
Parameters of recurrence quantification analysis. (1) – 400–0 ms, (2) 0–500 ms, (3) 500–1000 ms, and (4) 1000–1600 ms.

Recurrence plots and the corresponding RQA parameters of ERP signals stimulated by ridged texture are shown in Figures 6, 7.

Caused by characteristic behavior of the phase space trajectory, an RP exhibits characteristic large-scale and small-scale patterns, which are caused by typical dynamical behavior (Marwan et al., 2007). Typical small-scale patterns are single dots, diagonal lines and vertical/horizontal lines. Large scale patterns can be characterized as homogenous, periodic, drift and disrupted, which gives hints about the dynamics of the system (Marwan, 2003). In Figure 6, the large-scale patterns of RPs showed an evolution of “homogenous–disrupted-homogenous-drift”.

As shown in Figures 6, 7, before touching (−400–0 ms), the RPs show a homogenous state with uniform distribution of black areas. All RQA parameters were small. This result indicated that the EEG system was stable and fluctuated over a small range during this state. During the touching process (0–1000 ms), there were two states. Within 0 ∼ 500 ms, there was no obvious regularity in the patterns of RPs. The black and white area distributions were relatively concentrated, and the white area predominates, indicating RPs show a disrupted state. RPs suggested that in this state the change of EEG signals was significant; the EEG system presented drastic non-stationarity and fluctuations. All RQA parameters increased significantly. This stage corresponded just to the production stage of the P100, P200, and P300 component. However, in the range of 500–1000 ms, RPs presented a homogenous state with uniform distribution of black areas and the RQA parameters decreased, indicating that the tactile perception had been completed in this stage. After touching (1000–1600 ms), the RPs of EEG signals shows a drifted pattern and the distribution of the black areas was relatively uniform and mainly along the direction parallel to LOI, indicating that the EEG signals change slowly with time in this stage and the fluctuation was relatively stable. Compared with the homogenous state, the values of RQA increased.

In the RPs of three samples, the differences focused in the area of 1000–1600 ms. Though the black areas mainly paralleled to LOI, the RPs of the flat shape sample were sparse, indicating in the drift process that compared with other samples, the fluctuation of EEG signals were larger.

RPs visualized the recurrence behavior of the non-linear EEG signal; however, they did not quantify the dynamic characteristics of the EEG signal. The different RQA parameters showed different dynamic meanings of the system. For example, *ENT* reflects the complexity of the diagonal length distribution in the RPs and a higher value indicates a more complex system; *L* and *TT* both describe the relative speed of the system state change and describe the stability of the system.

By comparing the RPs and RQA of EEG signals excited by the three texture samples, it can be found that with the change of the recurrence mode from homogenous state to disrupted state to homogenous state to drift state, RQA parameters showed a trend of increasing – decreasing – increasing. During the tactile perception process (0–1000 ms), it was found that all RQA parameters represented a regulation of flat shape > round shape > sharp shape except the parameters of *L*. These results suggested that the flat shape texture with complex geometric structure tended to evoke high RQA parameters and induce the brain EEG system to a strong non-stationary and fluctuating state.

## Conclusion

In this study, the electrical activity of the brain evoked by the tactile perceptions of ridged textures was investigated using ERP and non-linear methods. The components of ERP evoked during the perceptual processing of texture shape were investigated. The non-linear dynamic behavior of EEG systems related with tactile perception was quantitatively characterized by RPs and RQA. The conclusions are as follows.

The sensation of mechanical stimulation occurred primarily in the parietal lobe of the contralateral brain. The early component of P100 and P200, and the late component of P300 of ERP were evoked during perceiving different texture shape.

The shape features affect electrical activity of brain during the tactile perceptions. The latency of P100 induced by sharp texture was significantly faster and the amplitude was significantly larger than others, indicating the sharp feature of texture promoted the neurotransmission, which in-turn enhanced the neuronal response properties and temporal processing. The latency of P200 induced by flat texture was significantly faster and the amplitude was significantly larger than others, indicating the complex geometric structure can also enhanced the neuronal response properties and temporal processing. The amplitude of P300 evoked by round shape texture was significantly lower than others, indicating the sharp feature and complex feature can be sensed easily and need less attentional resources in the tactile perception judgment.

During the whole tactile perception, RPs showed an evolution of “homogenous–disrupted – homogenous – drift” and the corresponding RQA parameters show a trend of increasing – decreasing – increasing. The large-scale structures of RP and RQA parameters can visualize and quantize the evolutionary regulation of the dynamic behavior of the EEG system along with the tactile process. The flat texture with complex geometric structure tended to evoke high RQA parameters and induced the brain EEG system to a strong non-stationary state. RPs and RQA provide protential methods for feature extraction and state recognition of EEG signal evoked by textured surface during tactile perception.

In this study, we just studied the effects of edge shape of triangular ridges on tactile perception. More work about ridge features can be done to establish their effects on tactile perception, for example the ridge profiles (rectangular and sinusoidal, etc.), ridge properties (height, width, and density, etc.) as well as other physical materials (metal and plastic, etc.). The number of subjects was limited and a larger number of subjects which is more than 20 is expected in the future study. The related tactile friction studies can be carried out to optimize surface tactile characteristics on products. More non-linear method such as correlation dimension, Lyapunov index, and wavelet entropy analysis can be discussed in further study.

## Data Availability Statement

The raw data supporting the conclusions of this article will be made available by the authors, without undue reservation.

## Ethics Statement

The studies involving human participants were reviewed and approved by Xuzhou Central Hospital Medical Ethics Committee. The patients/participants provided their written informed consent to participate in this study.

## Author Contributions

WT, SB, MZ, and GC: study conception and design. RL, GC, YP, SC, YS, and CH: acquisition of data. WT, MZ, RL, and SB: drafting of the manuscript. All authors: analysis and interpretation of data, and review the manuscript.

## Conflict of Interest

The authors declare that the research was conducted in the absence of any commercial or financial relationships that could be construed as a potential conflict of interest.
